# From Clinical Trial Efficacy to Real-World Effectiveness and Safety: Two-Year Outcomes of Cladribine in Relapsing—Remitting Multiple Sclerosis

**DOI:** 10.3390/jcm15135121

**Published:** 2026-07-01

**Authors:** Duygu Özkan Yaşargün, Recai Türkoğlu

**Affiliations:** Department of Neurology, Haydarpaşa Numune Training and Research Hospital, University of Health Sciences, İstanbul 34668, Turkey

**Keywords:** cladribine, relapsing-remitting multiple sclerosis, real-world study, NEDA-3, lymphopenia, safety, effectiveness

## Abstract

**Background/Objectives:** Cladribine is a high-efficacy immune reconstitution therapy for relapsing multiple sclerosis, but additional real-world data remain valuable, particularly from single-center cohorts with combined clinical, radiological, and laboratory follow-up. This study aimed to evaluate the 24-month effectiveness and safety of cladribine in patients with relapsing-remitting multiple sclerosis (RRMS), with a particular focus on no evidence of disease activity (NEDA-3) and lymphopenia. **Methods:** This retrospective single-center observational cohort study included adults with RRMS treated with cladribine who had at least 24 months of clinical and MRI follow-up. NEDA-3 was defined as the absence of clinical relapses, disability progression, and MRI activity. Brain MRI was performed at 6, 12, and 24 months. Absolute lymphocyte counts were monitored longitudinally, and lymphopenia was graded according to standard criteria. **Results:** A total of 71 patients were included. NEDA-3 was achieved in 61 patients (85.9%) at 12 months and 50 patients (70.4%) at 24 months. No demographic or baseline clinical variable was independently associated with NEDA-3 at either time point. Lymphopenia occurred in 53 patients (74.6%), including grade 1 in 14 (19.7%), grade 2 in 23 (32.4%), grade 3 in 14 (19.7%), and grade 4 in 2 (2.8%). One patient developed herpes zoster infection during follow-up. **Conclusions:** Cladribine demonstrated durable effectiveness and a manageable safety profile over 24 months in a real-world RRMS population. The close alignment between our findings and those reported in pivotal clinical trials underscores the external validity of cladribine efficacy beyond controlled study environments. These data provide valuable real-world evidence bridging the gap between controlled research settings and everyday patient care, thereby strengthening the evidence base for cladribine in contemporary MS management.

## 1. Introduction

Multiple sclerosis (MS) is a chronic immune-mediated disease of the central nervous system characterized by inflammatory demyelination and progressive neurological damage, which may lead to irreversible disability over time [1,2,3]. Cladribine tablets have emerged as a highly effective treatment option for relapsing multiple sclerosis and are considered an immune reconstitution therapy [2,4,5,6,7,8,9,10,11]. Through selective targeting of lymphocytes, cladribine induces immune modulation and has shown beneficial effects on clinical and radiological disease activity [2,5,12].

The efficacy of cladribine has been demonstrated in pivotal clinical studies. In the CLARITY trial, cladribine significantly reduced annualized relapse rates, lowered the risk of disability progression, and reduced MRI measures of disease activity compared with placebo [5]. MRI analyses from CLARITY further showed significant reductions in T1 gadolinium-enhancing, active T2, and combined unique lesions [13]. In addition, post hoc analysis of CLARITY demonstrated that sustained freedom from disease activity, defined by the absence of relapses, disability progression, and MRI activity, was significantly more frequent with cladribine than with placebo [6]. Extension and long-term follow-up studies also suggested durable clinical and MRI benefits after the initial treatment period [7,9,14].

Although randomized trials established the efficacy of cladribine, real-world data are still needed to better define its effectiveness and safety in routine clinical practice. Observational studies from different clinical settings have generally confirmed reductions in relapse activity, favorable disability outcomes, high rates of NEDA-3 retention, and manageable safety profiles [4,11,12,15,16,17,18]. However, additional single-center real-world data remain valuable, particularly for combined clinical, radiological, and laboratory follow-up.

Therefore, in this retrospective single-center study, we aimed to evaluate the 24-month clinical, radiological, and laboratory outcomes of cladribine treatment in patients with relapsing-remitting multiple sclerosis, with a particular focus on NEDA-3 status and lymphopenia during follow-up.

## 2. Methods

### 2.1. Study Design and Participants

This study was conducted as a retrospective, single-center observational cohort study evaluating the clinical, laboratory, and radiological outcomes of patients with relapsing-remitting multiple sclerosis (RRMS) treated with cladribine. Cladribine was an escalation/switch therapy in patients who had previously received one or more disease-modifying therapies, mainly because of ongoing disease activity, suboptimal response, tolerability issues, or the need for a higher-efficacy treatment with a convenient dosing schedule, as determined by the treating neurologist. Patients receiving cladribine therapy between January 2022 and December 2025 at the Multiple Sclerosis Unit of our institution were retrospectively identified through the hospital electronic medical record system. Among these patients, only those who had completed at least 24 months of clinical and MRI follow-up after cladribine treatment initiation were included in the final analysis. The data were gathered retrospectively from the institutional electronic medical records and the iMed platform for MS data entry (MSBase Foundation, Melbourne, VIC, Australia). All variables were recorded as documented by the treating neurologist. Before data export, the study team manually updated the iMed records to minimize incomplete entries. All available clinical, laboratory, and neuroimaging data were reviewed for a follow-up period of 24 months after treatment initiation.

Eligible participants were adults aged older than 18 years who had a confirmed diagnosis of relapsing-remitting multiple sclerosis (RRMS) according to the 2017 revised McDonald criteria [19] and who received cladribine therapy with at least two years of available clinical and MRI follow-up data. Patients with secondary progressive multiple sclerosis (SPMS), primary progressive multiple sclerosis (PPMS), a history of malignancy, or incomplete clinical or radiological follow-up were excluded from the study.

### 2.2. Treatment Protocol

Cladribine was administered according to the following standard treatment schedule. The treatment consisted of four short courses over two years, followed by observation without further drug administration. In year 1, patients received two treatment courses administered in month 1 and month 2, and in year 2, two additional courses were given in month 12 and month 13. Each treatment course consisted of oral cladribine tablets administered once daily for 4–5 consecutive days, with the exact number of tablets determined according to the patient’s body weight. The total cumulative dose was 3.5 mg/kg, administered as 1.75 mg/kg in year 1 and 1.75 mg/kg in year 2.

### 2.3. Clinical Evaluation

Neurological disability was assessed using the Expanded Disability Status Scale (EDSS). EDSS scores were recorded at baseline and during routine clinical follow-up visits for 24 months. The Multiple Sclerosis Severity Score (MSSS) was calculated using baseline EDSS scores and disease duration according to the original algorithm described by Roxburgh et al. [20]. Clinical relapse events occurring during the follow-up period were identified through medical records and outpatient visit documentation. Oligoclonal band (OCB) patterns at disease onset were classified according to the conventional five-type classification system. Type 1 was defined as the absence of OCBs in both cerebrospinal fluid (CSF) and serum; Type 2 as CSF-restricted OCBs; Type 3 as CSF-restricted OCBs with additional identical bands in serum; Type 4 as identical OCBs in CSF and serum, also referred to as a mirror pattern; and Type 5 as a monoclonal pattern in both CSF and serum.

### 2.4. Laboratory and Radiological Monitoring

Routine laboratory evaluations were performed as part of standard clinical follow-up. Absolute lymphocyte counts were recorded at baseline and during follow-up visits. Brain magnetic resonance imaging (MRI) was performed according to routine clinical practice at 6, 12, and 24 months after treatment initiation. Radiological disease activity was assessed based on the presence of new or enlarging T2-hyperintense lesions and gadolinium-enhancing lesions on T1-weighted sequences. MRI findings were reviewed using the official radiology data available in the hospital information system.

### 2.5. Outcome Measures

No evidence of disease activity (NEDA-3) was defined as the absence of clinical relapses, disability progression, and MRI activity during the follow-up period. Clinical relapse was defined as the occurrence of a new neurological attack during clinical follow-up. Disability progression was evaluated using the Expanded Disability Status Scale (EDSS) and was defined as a confirmed increase of ≥1.0 point in patients with a baseline EDSS ≤5.5 or ≥0.5 points in those with a baseline EDSS >5.5. MRI activity was defined as the presence of new or enlarging T2 lesions or gadolinium-enhancing lesions on follow-up MRI. Patients were considered to have achieved NEDA-3 when all three criteria were absent during the study period. In addition, changes in absolute lymphocyte counts were evaluated every at following months: 1, 3, 6, 9, 12, 13, 15, 18, 21, 24. Lymphopenia was defined as follows: Grade 1, ≥0.8–<1.0 × 10^9^/L; Grade 2, ≥0.5–<0.8 × 10^9^/L; Grade 3, ≥0.2–<0.5 × 10^9^/L; and Grade 4, <0.2 × 10^9^/L.

### 2.6. Statistical Analysis

Statistical analyses were performed using IBM SPSS Statistics software (Version 21.0, IBM Corp., Armonk, NY, USA). Variables were presented as mean ± standard deviation, median (range), or number (percentage), where appropriate. Continuous variables were assessed for normality using graphical methods and hypothesis tests. Continuous variables with normal distribution were compared using student t test for independent samples and Mann–Whitney U test was used to compare continuous variables without normal distribution. For statistical analyses, OCB pattern was dichotomized as Type 2 versus non-Type 2 because Type 2 was the most frequent pattern and the numbers of patients in the other categories were limited. Associations between categorical variables were evaluated using chi-square or Fisher’s exact tests. To identify factors associated with treatment response, univariable analyses were initially performed to explore potential predictors of achieving NEDA-3 status at follow-up. Variables considered clinically relevant were subsequently included in multivariable logistic regression models to determine independent predictors of NEDA-3 status. The final multivariable logistic regression models included age, sex, OCB pattern at disease onset, comorbidity status, family history of MS, disease duration, prior treatment category, and baseline EDSS score. Results were reported as odds ratios (ORs) with 95% confidence intervals (CIs) and *p*-values. A two-sided *p*-value of less than 0.05 was considered statistically significant.

## 3. Results

### 3.1. Patient Characteristics

A total of 83 patients receiving cladribine therapy were initially screened. Of these, 12 patients were excluded because they had not yet completed 24 months of clinical and MRI follow-up. Therefore, 71 patients were included in the final analysis. Table 1 shows demographic and clinical characteristics of patients. Overall, NEDA-3 status was achieved in 61 patients (85.9%) at 12 months and 50 patients (70.4%) at 24 months. Only Types 1–4 OCB patterns were observed.

The prior disease-modifying therapies used before cladribine included fingolimod in 42 patients (59.2%), interferon beta-1a in 22 (31.0%), interferon beta-1b in 21 (29.6%), teriflunomide in 17 (23.9%), glatiramer acetate in 15 (21.1%), dimethyl fumarate in 13 (18.3%), ocrelizumab in 7 (9.9%), natalizumab in 5 (7.0%), azathioprine in 5 (7.0%), peginterferon beta-1a in 2 (2.8%), and alemtuzumab in 2 (2.8%). Overall, 11 patients (15.5%) had received a single prior drug, whereas 60 patients (84.5%) had received multiple prior drugs, including two prior drugs in 37 patients (52.1%), three prior drugs in 19 patients (26.8%), four prior drugs in 3 patients (4.2%), and five prior drugs in 1 patient (1.4%). Patients were switched to cladribine.

New MRI activity was observed in 6 patients (8.5%) during the 24-month follow-up period. The lesion locations were as follows: cerebellar vermis in one patient, C7–T1 spinal cord level in one patient, left occipital periventricular region in one patient, left ventral mesencephalon in one patient, right parietal region in one patient, and splenium of the corpus callosum in one patient.

During the overall 24-month follow-up period, 21 patients did not maintain NEDA-3 status. The contributing components were clinical relapse in 13 patients, MRI activity in 6 patients, and EDSS progression in 5 patients. Because NEDA-3 components may overlap within individual patients, overlapping components were observed in 3 patients, including 2 patients with both clinical relapse and MRI activity and 1 patient with both clinical relapse and EDSS progression.

### 3.2. Univariate and Multivariate Analysis of Factors for NEDA-3 Status at 12 Months

Univariate analyses were performed to evaluate potential factors associated with achieving NEDA-3 status at 12 months (Table 2). No significant associations were observed between NEDA-3 status and demographic or clinical characteristics, including age, sex, OCB pattern at disease onset, presence of comorbidities, family history of multiple sclerosis, duration of disease, prior treatment exposure, or baseline EDSS score.

Variables with potential clinical relevance were subsequently included in a multivariable logistic regression model to explore independent predictors of treatment response. However, none of the evaluated variables were found to be independently associated with achieving NEDA-3 at 12 months.

### 3.3. Univariate and Multivariate Analysis of Factors for NEDA-3 Status at 24 Months

A similar analysis was conducted to identify factors associated with NEDA-3 status at 24 months (Table 3). Univariate analyses did not demonstrate significant associations between NEDA-3 status and the evaluated demographic or clinical variables, including age, sex, OCB pattern at disease onset, comorbidity status, family history of multiple sclerosis, duration of disease, prior treatment history, or baseline EDSS score.

Subsequent multivariable logistic regression analysis likewise did not identify any independent predictors of achieving NEDA-3 status at 24 months.

Complete multivariable logistic regression results are provided in Table 4. In the final models including age, sex, OCB pattern at disease onset, comorbidity, family history of MS, disease duration, prior treatment category, and baseline EDSS, no variable was independently associated with NEDA-3 status at either 12 or 24 months.

The multivariable models included age, sex, OCB pattern at disease onset, comorbidity, family history of MS, disease duration, prior treatment category, and baseline EDSS. For categorical variables, the following reference categories were used: female sex, non-Type 2 OCB pattern at disease onset, absence of comorbidity, absence of family history of MS, and monotherapy as prior treatment. No variable was independently associated with NEDA-3 status at either 12 or 24 months.

### 3.4. Side Effects

**Figure 1:**
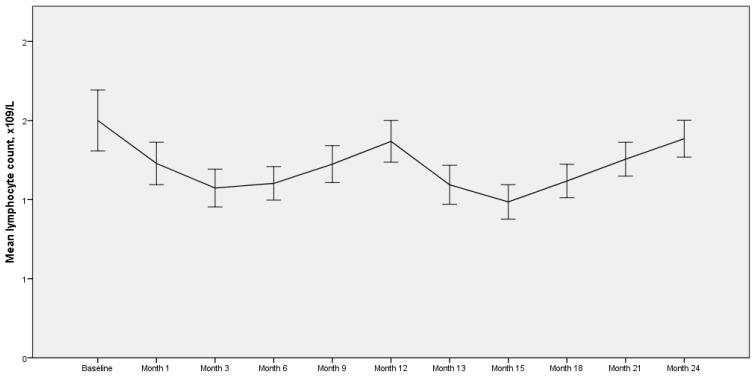
Longitudinal trend of absolute lymphocyte counts during 24-month follow-up. Error bars indicate 95% confidence intervals.

Longitudinal absolute lymphocyte count trends during follow-up are shown in Figure 1. During the 24-month follow-up period, lymphopenia was observed in the majority of patients. Based on the predefined grading criteria (Grade 1: ≥0.8–<1.0 × 10^9^/L; Grade 2: ≥0.5–<0.8 × 10^9^/L; Grade 3: ≥0.2–<0.5 × 10^9^/L; Grade 4: <0.2 × 10^9^/L), lymphocyte counts remained within the normal range in 18 patients (25.4%), while 53 patients (74.6%) developed lymphopenia at some point during follow-up.

Among these, Grade 1 lymphopenia occurred in 14 patients (19.7%), Grade 2 in 23 patients (32.4%), Grade 3 in 14 patients (19.7%), and Grade 4 in 2 patients (2.8%). Overall, moderate lymphopenia (Grade 2) was the most frequently observed category.

In addition, one patient developed herpes zoster infection during the follow-up period.

## 4. Discussion

In this retrospective single-center cohort of patients with RRMS treated with cladribine, high rates of disease control were observed over 24 months. NEDA-3 was achieved in 85.9% of patients at 12 months and 70.4% at 24 months. In addition, no demographic or baseline clinical variable was independently associated with NEDA-3 status at either time point. From a safety perspective, lymphopenia was the most frequent adverse event, occurring in 74.6% of patients, while grade 3 and grade 4 lymphopenia were observed in 19.7% and 2.8% of patients, respectively. Only one patient developed herpes zoster during follow-up. Taken together, these findings support the effectiveness of cladribine in routine clinical practice and confirm a safety profile consistent with previous clinical trials and real-world studies [4,5,7,12,16,21].

The efficacy outcomes observed in our cohort are broadly in line with previously published real-world data. In the Belgian single-center cohort reported by Aerts et al., 72.6% of patients retained NEDA-3 status at a mean follow-up of 22.6 months, with relapses, disability progression, and brain MRI activity occurring in 16.7%, 8.5%, and 6.3% of patients, respectively [4]. Similarly, Petracca et al. reported NEDA-3 in 64% of patients at a median follow-up of 22 months in a multicenter real-world study [21]. In the Danish nationwide cohort, cladribine treatment was associated with a marked reduction in annualized relapse rate after treatment initiation, further supporting its effectiveness in routine practice [12]. The Qatar real-world study also demonstrated strong suppression of relapse and MRI activity, with no relapses in year 2 and high proportions of patients remaining free of gadolinium-enhancing lesions during follow-up [16]. In the Australian MSBase registry substudy, cladribine was associated with a disease-modifying effect even in a cohort characterized by older and more disabled patients, with approximately 80% remaining free from EDSS progression and 65% remaining relapse-free after 2 years [17]. Although our 24-month NEDA-3 rate appears numerically favorable, these comparisons should be interpreted with caution because study populations, follow-up duration, MRI schedules, and definitions of treatment baseline were not identical across cohorts.

One of the notable findings of our study was the absence of significant predictors of NEDA-3 at either 12 or 24 months. This differs from the multicenter study by Petracca et al., in which a higher number of previous treatments was associated with lower probability of retaining NEDA-3, baseline MRI activity predicted follow-up MRI activity, and higher relapse rate in the year before cladribine initiation was associated with sustained disability worsening [21]. In the Belgian cohort, disease activity during the first year after cladribine initiation tended to occur more frequently in patients with at least two prior DMTs and in those switching from fingolimod, although these trends did not reach statistical significance [4]. The lack of significant predictors in our cohort may reflect the relatively limited sample size and number of outcome events, which may have reduced statistical power to detect modest associations. It is also possible that the cohort was relatively homogeneous regarding treatment selection and follow-up.

Our safety findings were also consistent with the established profile of cladribine. In the pivotal CLARITY trial, lymphocytopenia and herpes zoster were more frequent in the cladribine groups than in the placebo group [5]. In CLARITY Extension, grade ≥3 lymphopenia remained more common in cladribine-treated patients, although grade 4 lymphopenia was infrequent and most patients recovered to grade 0–1 lymphopenia by study end [7]. In the Qatar cohort, grade 3 lymphopenia occurred in around 11% of the patients [16]. Likewise, the Danish nationwide study found that adverse events were generally mild or moderate, and herpes zoster was reported in only two patients [12]. In our cohort, lymphopenia was common but severe infectious complications were rare, and only one case of herpes zoster was observed. These findings suggest that, in routine practice, cladribine-associated lymphopenia is frequent but usually clinically manageable.

An important issue when interpreting our findings is the methodological heterogeneity among real-world studies. In our cohort, MRI follow-up was performed at 6, 12, and 24 months according to routine clinical practice, and NEDA-3 was assessed across the full observation period from treatment initiation. In contrast, some real-world studies, including the Belgian and Danish cohorts, applied a 3-month re-baseline approach for efficacy analyses [4]. Such methodological differences may be relevant because they may influence reported NEDA-3 rates and limit direct numerical comparisons across studies. Therefore, our results are best interpreted as further support for the real-world effectiveness of cladribine rather than as evidence of superiority over other cohorts.

This study has several strengths, including the availability of 24-month clinical, radiological, and laboratory follow-up data and the combined evaluation of NEDA-3 and lymphopenia in a routine care setting. However, several limitations should also be acknowledged. First, the retrospective and single-center design limits generalizability. Second, the sample size was relatively modest, and the number of patients not achieving NEDA-3 was limited. Therefore, the multivariable logistic regression models may have been underpowered to detect modest but clinically relevant associations, and the absence of statistically significant predictors should be interpreted with caution because of the possibility of type II error. Third, the absence of a comparator group prevents direct assessment against alternative treatment strategies. Finally, MRI assessments were performed according to routine practice rather than a fully standardized research protocol, and cross-study comparisons are complicated by differences in baseline definitions and follow-up methodology.

## 5. Conclusions

Cladribine demonstrated durable effectiveness and a manageable safety profile over 24 months in a real-world RRMS population. The close alignment between our findings and those reported in pivotal clinical trials underscores the external validity of cladribine efficacy beyond controlled study environments. By confirming the reproducibility of these outcomes in an unselected population, our study provides valuable real-world evidence bridging the gap between controlled research settings and everyday patient care, thereby strengthening the evidence base for cladribine in contemporary MS management.

## Figures and Tables

**Table 1:** Demographic and clinical characteristics of the patients at baseline.

	*n* = 71
Age, y, mean ± SD, median (range)	45.2 ± 9.9, 45 (21–74)
Age at onset, y, mean ± SD, median (range)	37.1 ± 9.4, 38 (18–64)
Female sex	46 (64.8%)
Presence of comorbidity *	19 (26.8%)
Family history of MS	7 (9.9%)
OCB pattern at disease onset	
Type 1	20 (28.2%)
Type 2	42 (59.2%)
Type 3	7 (9.9%)
Type 4	2 (2.8%)
Prior treatment	
Single drug	11 (15.5%)
Multiple drugs	60 (84.5%)
Duration of disease, y, mean ± SD, median (range) †	8.1 ± 4.9, 7 (1–25)
EDSS score, mean ± SD, median (range)	3.4 ± 1.1, 3 (2–7)
MSSS, mean ± SD, median (range)	5.5 ± 2.0, 5.4 (1.6–9.7)

Unless otherwise stated, data presented as n (%). Abbreviations: MS, multiple sclerosis; EDSS, Expanded Disability Status Scale; OCB, oligoclonal band; MSSS, multiple sclerosis severity score. * comorbidities: hypertension, 7 patients; Hashimoto, 5 patients; inflammatory bowel disease, 2 patients; diabetes, 2 patients, † at baseline.

**Table 2:** Univariate analysis of factors for NEDA-3 status at 12 months.

Factor	NEDA-3 Not Achieved (n = 10)	NEDA-3 Achieved (n = 61)	*p*-Value
Age, y	45.2 ± 9.7	45.2 ± 10.0	0.993
Sex			
Male	4 (40.0%)	21 (34.4%)	0.733
Female	6 (60.0%)	40 (65.6%)	
OCB pattern at disease onset			
Non-Type 2	4 (40.0%)	25 (41.0%)	1.000
Type 2	6 (60.0%)	36 (59.0%)	
Comorbidity			
Absent	8 (80.0%)	44 (72.1%)	0.719
Present	2 (20.0%)	17 (27.9%)	
Family history			
Absent	8 (80.0%)	56 (91.8%)	0.254
Present	2 (20.0%)	5 (8.2%)	
Duration of disease, y *	6.1 ± 2.7	8.4 ± 5.1	0.198
Prior treatment			
Single drug	1 (10.0%)	10 (16.4%)	1.000
Multiple drugs	9 (90.0%)	51 (83.6%)	
Baseline EDSS	3.3 ± 0.8	3.4 ± 1.1	0.920

* at baseline. OCB, oligoclonal band.

**Table 3:** Univariate analysis of factors for NEDA-3 status at 24 months.

Factor	NEDA-3 Not Achieved (n = 21)	NEDA-3 Achieved (n = 50)	*p*-Value
Age	43.7 ± 11.5	45.9 ± 9.1	0.406
Sex			
Male	7 (33.3%)	18 (36.0%)	0.830
Female	14 (66.7%)	32 (64.0%)	
OCB pattern at disease onset			
Non-Type 2	7 (33.3%)	22 (44.0%)	0.404
Type 2	14 (66.7%)	28 (56.0%)	
Comorbidity			
Absent	17 (81.0%)	35 (70.0%)	0.341
Present	4 (19.0%)	15 (30.0%)	
Family history			
Absent	19 (90.5%)	45 (90.0%)	1.000
Present	2 (9.5%)	5 (10.0%)	
Duration of disease, y *	6.9 ± 4.0	8.6 ± 5.1	0.144
Prior treatment			
Single drug	3 (14.3%)	8 (16.0%)	1.000
Multiple drugs	18 (85.7%)	42 (84.0%)	
Baseline EDSS	3.3 ± 0.9	3.4 ± 1.1	0.759

* at baseline. OCB, oligoclonal band.

**Table 4:** Multivariable logistic regression analysis for NEDA-3 status at 12 and 24 months.

Variable	OR	95% CI	*p*-Value
**NEDA-3 status at 12 months**			
Age	0.964	0.886–1.049	0.394
Sex	0.826	0.180–3.784	0.805
OCB pattern at disease onset	1.200	0.244–5.893	0.822
Comorbidity	1.636	0.241–11.087	0.614
Family history of MS	0.444	0.060–3.313	0.429
Disease duration	1.191	0.949–1.494	0.131
Prior treatment	0.271	0.023–3.201	0.300
Baseline EDSS	1.371	0.606–3.102	0.448
**NEDA-3 status at 24 months**			
Age	1.000	0.940–1.063	0.997
Sex	1.067	0.339–3.357	0.912
OCB pattern at disease onset	0.693	0.219–2.194	0.532
Comorbidity	1.371	0.349–5.387	0.651
Family history of MS	1.292	0.205–8.136	0.785
Disease duration	1.097	0.951–1.265	0.203
Prior treatment	0.589	0.115–3.021	0.526
Baseline EDSS	1.118	0.652–1.917	0.685

Abbreviations: NEDA-3, no evidence of disease activity-3; OR, odds ratio; CI, confidence interval; MS, multiple sclerosis; EDSS, Expanded Disability Status Scale; OCB, oligoclonal band.

## Data Availability

The data presented in this study are available on request from the corresponding author. The data are not publicly available due to privacy and ethical restrictions.

## Abbreviations

The following abbreviations are used in this manuscript:

MS	Multiple sclerosis
RRMS	Relapsing-remitting multiple sclerosis
NEDA	No Evidence of Disease Activity
MRI	Magnetic Resonance Imaging
IMED	MSBase Foundation
PPMS	Primary progressive multiple sclerosis
SPMS	Secondary progressive multiple sclerosis
